# Addition of inulin to probiotic yogurt: Viability of probiotic bacteria (*Bifidobacterium bifidum*) and sensory characteristics

**DOI:** 10.1002/fsn3.2154

**Published:** 2021-01-28

**Authors:** Dalia G. Kamel, Ahmed R.A. Hammam, Khalid A. Alsaleem, Dina M. Osman

**Affiliations:** ^1^ Dairy Science Department Faculty of Agriculture Assiut University Assiut Egypt; ^2^ Dairy and Food Science Department South Dakota State University Brookings SD USA; ^3^ Department of Food Science and Human Nutrition College of Agriculture and Veterinary Medicine Qassim University Buraydah Saudi Arabia

**Keywords:** *Bifidobacterium bifidum*, inulin, probiotic yogurt, sensory properties

## Abstract

The objective of this work was to study the effect of different concentrations of inulin (0.2, 0.4, and 0.6%) on the viability of probiotic bacteria (*Bifidobacterium bifidum*) and sensory characteristics of probiotic yogurt. The yogurt was manufactured with *Lactobacillus delbruckii* ssp. *bulgaricus* (Lb), *Streptococcus thermophilus* (St), and *Bifidobacterium bifidum* (Bb). Raw milk was received, heated to 90°C, and divided into 4 aliquots portions. All portions were inoculated with 5.11 log cfu of Lb and St combined and 5 log cfu of Bb per kg of milk. The first portion was utilized as control (T1) while 0.2, 0.4, and 0.6% of inulin were added to the second (T2), third (T3), and fourth (T4) portions, respectively. All treatments were incubated at 40°C until a pH of 4.6 was reached. Subsequently, the yogurt was cooled and stored at 4°C for 16 days. Titratable acidity, total bacterial count (TBC), Bb count, yeast count, mold count, and sensory evaluation were determined during the storage. The results showed that the addition of inulin and the storage period have significant effects (*p* < .05) on the titratable acidity of the yogurt. The storage of control was ended after 8 days at 4°C due to the growth of molds on the surface of the samples. The TBC decreased (*p* < .05) over time in control from 8.28 to 7.97 log cfu/g. It was also decreased (*p* < .05) with increasing the concentration of inulin. However, the addition of inulin increased (*p* < .05) the viability of Bb during the storage, as well as, acted as an antimicrobial against molds in T2, T3, and T4. Additionally, there were no significant differences (*p* > .05) in the sensory evaluation of all treatments. We conclude that inulin can be utilized in the manufacturing of probiotic yogurt as a prebiotic, which, inturn, enhances the growth of Bb and increase the shelf‐life.

## Graphical abstract

Inulin can be used in making yogurt to increase the viability of probiotic bacteria, as well as, increase the nutritional value of the product.

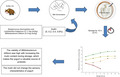

## 1 INTRODUCTION

Inulin is an oligosaccharide (fructan), soluble fiber in water, and can be extracted from several sources, including types of chicory, garlic, wheat, oat, and dahlia bulgur (Koruri et al., 2014). It has one β‐2‐1‐linked fructosyl unit with a terminal glucosyl unit (Paseephol et al., 2008). Inulin is considered a prebiotic and there is an interesting trend of using this ingredient to supplement fermented products, such as yogurt to improve gastrointestinal health, as well as, calcium absorption and boost the immune system. Additionally, it stimulates the growth of probiotic bacteria, such as *Lactobacillus* and *Bifidobacterium* in products during storage to reach the colon with a high viable number of those probiotics (Pereira et al., 2013).

Inulin has been utilized in many dairy products to improve the chemical, functional, and sensory characteristics, as well as the viability of probiotic bacteria in many dairy products, such as cheese (Buriti et al., 2007; Cardarelli et al., 2008; Hennelly et al., 2006; Salvatore et al., 2014; Zhang et al., 2021), yogurt (Canbulat & Ozcan, 2015; Li et al., 2019; Mazloumi et al., 2011; Sarwar et al., 2019; Shakerian et al., 2014), frozen yogurt (Isik et al., 2011; Rezaei et al., 2014), and ice cream (Akalın & Erişir, 2008; Akbari et al., 2016; Akın et al., 2007; Balthazar et al., 2018; Schaller‐Povolny & Smith, 1999; Tiwari et al., 2015).

Inulin has been used as a fat replacer to enhance the properties of low‐fat yogurt (Guven et al., 2005; Paseephol et al., 2008; Pimentel et al., 2013; Seydim et al., 2005). It has been found that the addition of 1% inulin improved the characteristics of low‐fat yogurt made from skim milk, which was similar to yogurt made from whole milk; however, higher amounts of inulin can lead to more whey separation (Guven et al., 2005). A similar study found that the firmness and brightness of low‐fat yogurt were improved with the addition of 2% inulin; yet, whey separation was also higher (Pimentel et al., 2013). Another study reported that incorporating inulin in low‐fat yogurt exhibited better rheological behavior that was similar to full‐fat yogurt, but it did not improve the firmness or apparent viscosity (Paseephol et al., 2008). Others found that the firmness of yogurt increased with the addition of 4% inulin as a prebiotic in yogurt (Oliveira et al., 2011). Also, inulin was utilized successfully to improve the texture of yogurt (Sarwar et al., 2019). Another study found that the overrun and glass transition of yogurt improved with the addition of 4 and 6% inulin while hardness was decreased (Muzammil et al., 2017). Furthermore, the addition of inulin at a range of 3 to 15% improved the functional characteristics with low syneresis (Żbikowska et al., 2020). Additionally, the apparent viscosity of yogurt increased with adding inulin up to 2% to yogurt (Helal et al., 2018). Another study reported that yogurt texture can be enhanced using inulin (Yi et al., 2010). The functional characteristics (e. g., overrun and meltability) of frozen yogurt enhanced with the addition of 2% inulin (Rezaei et al., 2014). Moreover, inulin has improved the functional characteristics of frozen yogurt, such as viscosity and meltability of frozen yogurt (Isik et al., 2011).

It has been found that inulin is enhancing the growth and viability of probiotic bacteria, such as *Saccharomyces boulardii*, *Lactobacillus rhamnosus*, and *Bifidobacterium animalis* in full‐fat yogurt (Canbulat & Ozcan, 2015; Sarwar et al., 2019; Shakerian et al., 2014); *Lactobacillus acidophilus* and *Lactobacillus delbrueckii* ssp. *bulgaricus* in low‐fat yogurt (Mazloumi et al., 2011); *Lactobacillus acidophilus* and *Bifidobacterium lactis*, as well as, lactic acid bacteria in frozen yogurt (Isik et al., 2011; Rezaei et al., 2014). Additionally, inulin has been utilized to encapsulate probiotic bacteria, such as *Lactobacillus acidophilus* and *Lactobacillus casei*, which resulted in maintaining a higher number of those bacteria at a rate of 7.0 log cfu/g (Krasaekoopt & Watcharapoka, 2014).

Some studies reported that inulin is improving the sensory characteristics of yogurt, while others found that it has no significant effects. It has been found that 1 or 2% of inulin did not result in significant differences in the sensory characteristics of low‐fat yogurt (Mazloumi et al., 2011). Another study did not report any effect of inulin on the sensory properties of ice cream made with fermented milk supplemented with inulin (Akın et al., 2007). On the other hand, it has been found that the addition of inulin and fructans improved the sensory characteristics of low‐fat stirred yogurt (Crispín‐Isidro et al., 2015). Inulin was found to increase the syneresis of probiotics to produce more volatile fatty acids that improve the sensory characteristics of yogurt up to 28 days of storage (Sarwar et al., 2019).

The addition of inulin in milk before making yogurt has a positive effect on the functional and sensory characteristics, as well as, consumer health because it maintains a high viable number of probiotics. As a result, the objectives of this study were to manufacture a probiotic yogurt supplemented with inulin (0.2, 0.4, and 0.6%) and studying the functional properties of this yogurt during storage at 4°C for 16 days.

## 2 MATERIAL AND METHODS

### 2.1 Manufacture of probiotic yogurt supplemented with inulin

Fresh buffalo's milk was obtained from the Animal Farm (Faculty of Agriculture, Assiut University, Assiut, Egypt), heated to 90°C for 5 min, and cooled to 40°C. The milk was then inoculated with 5.11 log cfu of *Lactobacillus delbruckii* ssp. *bulgaricus* (Lb) and *Streptococcus thermophilus* (St) (Dairy Science Department, Faculty of Agriculture, Assiut University, Egypt) combined, and 5 log cfu of *Bifidobacterium bifidum* (Bb) per kg of milk (Cairo MIRCEN, Faculty of Agriculture, Ain Shams University, Egypt). The milk was divided into 4 aliquots portions. The first portion was utilized as control (T1; with no inulin) while 0.2, 0.4, and 0.6% of inulin were added to the second (T2), third (T3), and fourth (T4) portions, respectively. All treatments were incubated at 40°C until a pH of 4.6 was reached and this process took approximately 4 hr. Subsequently, the yogurt was cooled and stored at 4°C for 16 days. This experiment was repeated 3 times using 3 different batches of raw milk.

### 2.2 Chemical and microbiological analyses

Titratable acidity was determined by calculating the lactic acid content in the yogurt (Akın et al., 2007; Sadler & Murphy, 2010). Total bacterial count (TBC), *Bifidobacterium bifidum* (Bb) count, yeast, and mold counts were determined as described by Hamdy and others (Hamdy et al., 2020). The chemical and microbiological analyses were performed at 0, 4, 8, 12, and 16 days.

### 2.3 Sensory evaluation

Sensory evaluation of probiotic yogurt was also determined as described by Hamdy and others with some modifications (Hamdy et al., 2020). Samples were evaluated for color and appearance (15 points), flavor (45 points), acidity (10 points), body and texture (30 points) to have 100 points as a total. The sensory characteristics were determined at 0 and 16 days.

### 2.4 Statistical analysis

Data were statistically analyzed using R software (R x64‐3.3.3, 9,205 NW 101st St, Miami, Florida, United States) by ANOVA using a GLM for each variable to study the effect of inulin and time or their interaction on the characteristics of probiotic yogurt. Mean separation was done using the least significant difference (LSD) comparison test when significant differences were detected at *p* < .05.

## 3 RESULTS AND DISCUSSION

### 3.1 Titratable acidity (% lactic acid)

**FIGURE 1:**
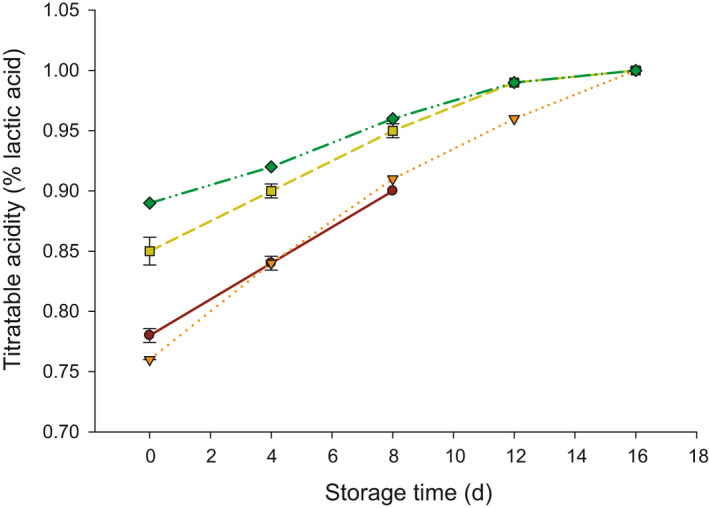
Acidity (%) of probiotic yogurt made with 0.0 (●), 0.2 (▼), 0.4 (■), and 0.6 (♦) % inulin

The effect of inulin on titratable acidity (%) of probiotic yogurt during 16 days of storage at 4°C is shown in Figure 1. Also, Table 1 is presented mean squares and *p*‐values that resulted from ANOVA. The addition of inulin increased the acidity of probiotic yogurt significantly (*p* < .05), and this increase was noticeable up to 12 days of storage. Additionally, storage time as well as the interaction of inulin and storage time significantly affected (*p* < .05) the acidity of probiotic yogurt (Table 1). The acidity was 0.78% in control (without inulin) at 0 day, and this value elevated (*p* < .05) to 0.9% after 8 d of storage. The control samples were excluded from the experiment after 8 d of storage since they were molded, which means less shelf‐life as compared to inulin treatments. During 16 days of storage, acidity of probiotic yogurt increased (*p* < .05) from 0.76% to 1%, 0.85 to 1%, and 0.89 to 1% when 0.2, 0.4, and 0.6% of inulin were added. After 16 days of storage, acidity was similar in all treatments.

**TABLE 1:** Mean squares and P‐values (in parentheses) of acidity (%), total bacterial count (TBC), and *Bifidobacterium bifidum* (Bb) count of probiotic yogurt made with 0.0, 0.2, 0.4, and 0.6% inulin

Factor	*df*	Acidity	TBC	Bb
Treatment^a^	3	0.028(<2.2e−16)^***^	30.04(<2e−16)^***^	24.69(<2.2e−16)^***^
Time^b^	4	0.047(<2.2e−16)^***^	6.72 (<2e−16)^***^	2.13(<2.2e−16)^***^
Replication	2	0.00002(0.6195)	0.013(0.41)	0.08(<4.02e−06)^***^
Treatment × Time	10	0.0016(7.054e−15)^***^	1.18(<2e−16)^***^	0.67(<2.2e−16)^***^
Error	34	0.000046	0.014	0.004

^a^ Treatment = 0, 0.2, 0.4, and 0.6% inulin

^b^ Time = 0, 4, 8, 12, and 16 days

* Statistically significant at *p* < .05

It was expected that the acidity of yogurt will be increased during the storage due to the growth of starter cultures. Similar trends were reported by other studies related to the effect of inulin on the acidity of yogurt during storage (Guven et al., 2005). Increasing the inulin from 0.2% to 0.6% led to elevating the titratable acidity of probiotic yogurt during storage. It was reported that increasing the percentage of inulin in frozen yogurt resulted in elevating the titratable acidity (Akın et al., 2007; Rezaei et al., 2014). This increase contributed to the high metabolism activity of starter cultures with increasing the inulin, which in turn, elevates the acidity development (Akın et al., 2007). However, this was not in agreement with other studies that increasing the inulin in yogurt is decreasing the acidity (Balthazar et al., 2016; Helal et al., 2018; Żbikowska et al., 2020). This can be related to the metabolism activity of starter cultures used to make yogurt.

### 3.2 Total bacterial count (TBC)

**FIGURE 2:**
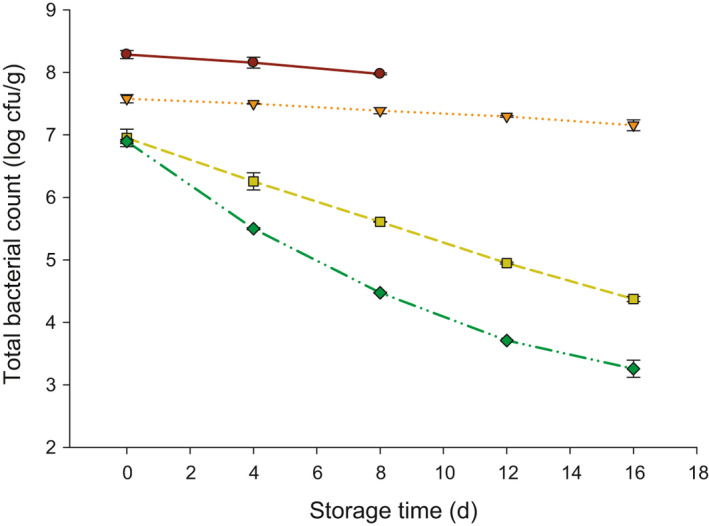
Total bacterial count (TBC; log cfu/g) in probiotic yogurt made with 0.0 (●), 0.2 (▼), 0.4 (■), and 0.6 (♦) % inulin

The TBC of probiotic yogurt made with inulin is shown in Figure 2. Table 1 is also exemplified the mean squares and P‐values of TBC. The addition of inulin (0.2, 0.4, and 0.6%), storage time, and their interaction showed a significant effect (*p* < .05) on the TBC of probiotic yogurt. The shelf‐life of control or non‐inulin yogurt samples was finished at 8 days since the molds were noticeable on the surface of yogurt. As a result, the last reading of TBC in control samples was recorded at 8 days. However, the TBC was decreased in all yogurt samples during storage at 4°C. The TBC in yogurt with no added inulin decreased from approximately 8.3 to 8 log cfu/g after 8 days. The TBC was lower at 0 day in yogurt with inulin as compared to control. The TBC at 0 d was found by 7.58, 6.95, and 6.89 log cfu/g when 0.2, 0.4, and 0.6% inulin added to the probiotic yogurt, respectively, and these values decreased to 7.15, 4.37, and 3.25 log cfu/g, respectively, after 16 days of storage.

Increasing the inulin content resulted in low TBC in probiotic yogurt, which refers to that inulin could have a preservative impact on growing other microorganisms, such as, St, Lb, yeast, and molds. Also, we theorized that the high lactic acid content (high acidity) in probiotic yogurt with inulin made the products non suitable for the growth of those microorganisms (act as a preservative), which reduced the TBC. It has been reported that the lactic acid resulted from the fermentation of lactose in milk acted as a preservative for the product (Delavari et al., 2014; Hekmat & Reid, 2006). Another reason is that incorporation of Bb starter cultures adversely affects the Lb counts, which is contributed to decreasing the TBC during storage (Baig & Prasad, 1996).

### 3.3 *Bifidobacterium bifidum* (Bb) count

**FIGURE 3:**
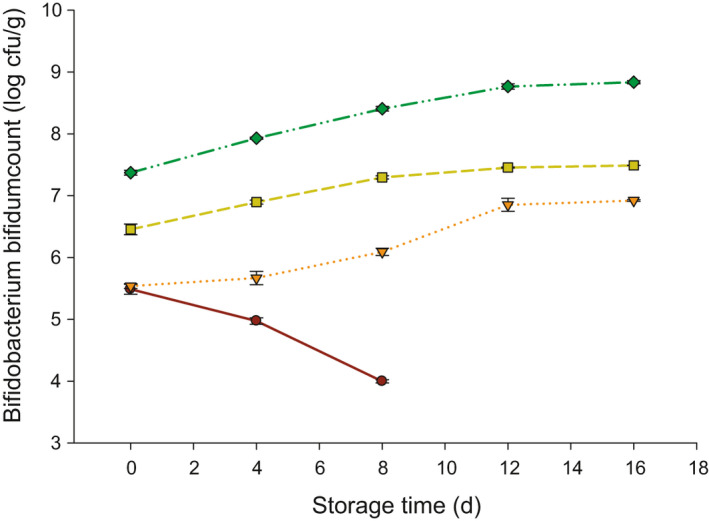
*Bifidobacterium bifidum* (Bb) count (log cfu/g) in probiotic yogurt made with 0.0 (●), 0.2 (▼), 0.4 (■), and 0.6 (♦) % inulin

The Bb count in probiotic yogurt is presented in Figure 3. The mean squares and P‐values of Bb count are also exemplified in Table 1. The addition of inulin, storage time, as well as their interaction has a significant effect (*p* < .05) on the Bb count of the probiotic count. Additionally, there was a replicate effect on the Bb count and this can be due to the slight difference in the count of Bb starter cultures during making the yogurt. The trend of Bb in control or T1 (no inulin added) was opposite to other treatments. The Bb in control decreased from 5.48 to 3.99 log cfu/g after 8 days of storage (end of storage for control due to growth of molds). However, this count increased from 5.53 to 6.92 log cfu/g in T2 (0.2% inulin), 6.45 to 7.49 log cfu/g in T3 (0.4% inulin), and 7.37 to 8.83 log cfu/g in T4 (0.6% inulin) after 16 days of storage at 4°C.

Increasing the inulin content in the probiotic yogurt enhanced the growth of Bb during the storage, which referred to that inulin is a suitable nutrient for Bb. Many studies reported similar findings. It has been reported that the viability of probiotic bacteria, such as *Lactobacillus acidophilus* and *Lactobacillus delbrueckii* ssp. *Bulgaricus,* increased with the addition of inulin in low‐fat yogurt (Mazloumi et al., 2011). Li and others have also reported that there a slight increase in starter cultures utilized to make low‐fat yogurt in the existence of 0.5% inulin (Li et al., 2019). Another study found that inulin maintained > 6.0 log cfu/g viable counts of *Saccharomyces boulardii* in yogurt (Sarwar et al., 2019). Adding 2% of inulin to probiotic yogurt led to increase the viability of *Lactobacillus rhamnosus* to 6.7 log cfu/g (Canbulat & Ozcan, 2015). Moreover, using inulin by 1% in yogurt enhanced the growth of *Bifidobacterium animalis* in probiotic yogurt with low proteolysis levels (Shakerian et al., 2014). Moreover, the viable counts of *Lactobacillus acidophilus* and *Bifidobacterium lactis* were increased when 2% of inulin added to frozen yogurt (Rezaei et al., 2014). It has also found that addition of inulin to frozen yogurt maintained a higher number of lactic acid bacteria that ranged from 8.1 to 8.5 during 3 months of shelf‐life (Isik et al., 2011). Furthermore, inulin has been utilized to produce fermented yogurt that was eventually used in making ice cream formulations to improve the viability of probiotic bacteria. It has been found that the addition of inulin in fermented yogurt maintained high viability of probiotics in ice cream as compared to control (Akın et al., 2007). Inulin has also been utilized in fermented soy milk and resulted in > 9.0 log cfu/ml (Mishra & Mishra, 2018). Additionally, inulin has been utilized to encapsulate probiotic bacteria, such as *Lactobacillus acidophilus* and *Lactobacillus casei*, which resulted in maintaining a higher number of these bacteria (7.0 log cfu/g) (Krasaekoopt & Watcharapoka, 2014).

### 3.4 Yeast and mold count

**FIGURE 4:**
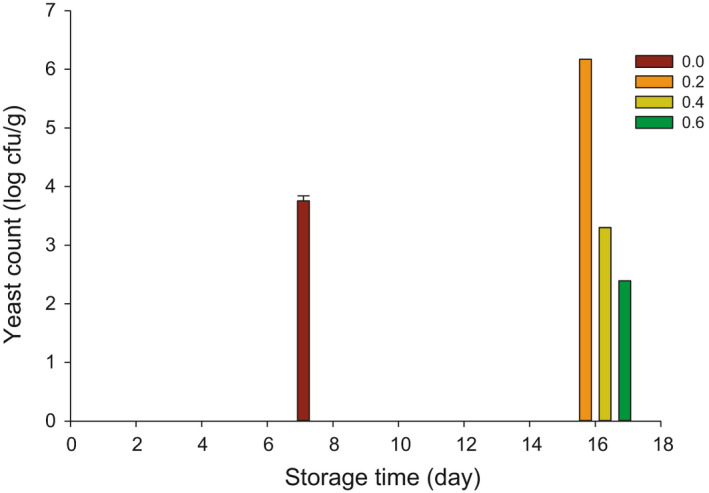
Yeast count (log cfu/g) in probiotic yogurt made with 0.0, 0.2, 0.4, and 0.6% inulin

The yeast count of probiotic yogurt is graphed in Figure 4. The yeast counts were not detected in control up to 8 days of storage, while it was detected in yogurt supplemented with inulin after 16 days of storage. The molds were detected in control after 8 days while yogurt supplemented with inulin did not exhibit any molds. The yeast count was 3.75 log cfu/g in control after 8 d. However, the count of yeast was 6.17, 3.3, and 2.39 log cfu/g after adding of 0.2, 0.4, and 0.6% inulin, respectively, after 16 days of storage at 4°C. It looks like the inulin has decreased the growth of yeast, which increases the shelf‐life or storage time of probiotic yogurt. Also, this preservative effect can be due to the high lactic acid content in those treatments (Delavari et al., 2014; Hekmat & Reid, 2006).

### 3.5 Sensory evaluation

The sensory evaluation of probiotic yogurt is presented in Table 2. The inulin does not affect (*p* > .05) the organoleptic characteristics of that yogurt, although there was a slight increase in the scores of flavor, texture, and appearance at 0 day as compared to control. The control was not judged at 16 d due to the short shelf‐life for that treatment that ended after 8 days. The overall scores tend to decrease after 16 d of storage and this might be due to the high acetaldehyde content produced from Bb starter cultures, which was not favorable to the panelists.

**TABLE 2:** Sensory evaluation of probiotic yogurt made with 0.0, 0.2, 0.4, and 0.6% inulin

Inulin (%)	Time (d)	Flavor (45)	Texture (30)	Appearance (15)	Acidity (10)	Overall score (100)
0.00	0	42.67	26.67	12.00	10.00	91.33
0.20		42.67	28.00	12.00	8.00	90.67
0.40		43.00	28.00	14.00	8.00	93.00
0.60		42.67	28.00	13.00	8.00	91.67
0.00	16	ND	ND	ND	ND	ND
0.20		41.33	28.00	13.00	8.00	90.33
0.40		42.00	28.00	13.00	8.00	91.00
0.60		40.33	27.70	12.00	8.00	88.00

Abbreviations: ND, not determined.

Other studies have reported similar results that inulin does not affect the sensory properties of probiotic yogurt. A study found that the addition of 1 or 2% of inulin did not result in significant differences in the sensory characteristics of low‐fat yogurt (Mazloumi et al., 2011). Akin and others also reported that inulin does not affect the sensory of ice cream made with fermented milk supplemented with inulin (Akın et al., 2007). However, others found that inulin can be utilized to improve the sensory characteristics of low‐fat yogurt. It has been reported that the addition of inulin and fructans improved the sensory properties of low‐fat stirred yogurt as compared to full‐fat yogurt due to forming gel on the casein micelles (Crispín‐Isidro et al., 2015). Using inulin has also been found to result in a smooth texture and good sensory characteristics (Seydim et al., 2005). Inulin increased the syneresis of probiotic to produce more volatile fatty acids that improve the sensory characteristics of yogurt up to 28 days of storage (Sarwar et al., 2019). The acceptability of low‐fat yogurt was increased when inulin was added (Pimentel et al., 2013), which was similar to full‐fat yogurt. Frozen yogurt was acceptable when 2% of inulin was added (Rezaei et al., 2014). The addition of inulin to yogurt improved the formation of acetaldehyde formation (Helal et al., 2018). Inulin in general improved the acceptance and flavor of yogurt (Canbulat & Ozcan, 2015). Furthermore, yogurt made from soy milk with inulin exhibited good sensory characteristics (Rinaldoni et al., 2012).

## 4 CONCLUSION

The addition of inulin as a prebiotic to probiotic yogurt improved the functional characteristics and the viability of *Bifidobacterium*, as well as increasing the shelf‐life of yogurt. Inulin has a potential health benefits, such as improving the absorption of calcium and enhancing gastrointestinal health. Inulin can have a promising application to supplement other dairy products, such as soft cheese, ice cream, and drinks.
